# SCHISTOSOMAL MYELORADICULOPATHY IN A NON-ENDEMIC AREA

**DOI:** 10.1590/1984-0462/2020/38/2018232

**Published:** 2020-01-13

**Authors:** Lívia Souza de Oliveira, Gabriela de Sio Puetter Kuzma, Luisa Carolina Vinhal Costa, Paulo Ramos David João

**Affiliations:** a Hospital Pequeno Príncipe, Curitiba. PR. Brazil.

**Keywords:** Schistosomiasis mansoni, Neuroschistosomiasis, Myelitis, Adolescent, Esquistossomose mansoni, Neuroesquistossomose, Mielite, Adolescente

## Abstract

**Objective:**

To report a schistosomal myeloradiculopathy case in a non-endemic area.

**Case description:**

A previously healthy 11-year-old boy, stricken by an acute loss of strength on his lower limbs, followed by a loss of strength on his upper limbs and upper body, associated with altered sensitivity of the vesical globe formation. The patient’s cerebrospinal fluid analysis showed eosinophilic meningitis, in addition to peripheral eosinophilia. The investigation resulted in a positive serology for *Schistosoma mansoni.* The treatment included steroids and praziquantel 60mg/kg, with a new dose after a month, as well as physical therapy for rehabilitation. The patient evolved with clinical improvement in the neurological exam, with a medullary section initially at C6, but now at T6. The patient is kept at prednisolone use (30mg/day) and longterm urinary catheter dependence.

**Comments:**

The schistosomiasis is endemic in many regions of Brazil; however, it has low incidence in the south of the country. Among its main manifestations, the schistosomal myeloradiculopathy is the most severe ectopic form of the disease, and should be suspected in patients with low back pain, strength and/or sensibility disorder of the lower limbs or urinary tract’s disturbance. Early diagnosis and treatment should be done in order to reduce severe neurological sequelae. Treatment includes schistosomiasis drugs, corticosteroids and/or surgery.

## RESUMO

**Objetivo:**

Relatar um caso de mielorradiculopatia esquistossomótica em área não endêmica.

**Descrição do caso:**

Paciente do sexo masculino, 11 anos, previamente hígido, com história aguda de paresia de membros inferiores, que evoluiu para membros superiores e tronco, associada à alteração de sensibilidade e formação de globo vesical. O exame do líquor demonstrava meningite eosinofílica, além de eosinofilia periférica. A investigação resultou em sorologia positiva para *Schistosoma mansoni.* O tratamento foi realizado com corticoterapia e praziquantel 60 mg/kg, com nova dose após um mês, além de fisioterapia para reabilitação. Evoluiu com melhora clínica no exame neurológico, com nível de secção medular que inicialmente correspondia a C6, encontrando-se atualmente em T6. Mantém uso de prednisolona 30 mg/dia e dependência de sonda vesical de demora.

**Comentários:**

A esquistossomose é uma doença endêmica em muitas regiões do Brasil, porém com pouca incidência no Sul do país. Dentre as principais manifestações, a mielorradiculopatia esquistossomótica é a forma ectópica mais grave e deve ser suspeitada na vigência de dor lombar, alteração de força e/ ou sensibilidade de membros inferiores e distúrbio urinário. O diagnóstico e o tratamento devem ser instituídos precocemente para diminuir o risco de sequelas neurológicas graves. O tratamento pode ser realizado com esquistossomicidas, corticosteroides e/ ou cirurgia.

## INTRODUCTION

Schistosomiasis is caused by a helmint of the genus *Schistosoma*. The transmission of the disease depends on whether the infected person is the definitive host, on whether water collection systems have inadequate sanitation and on whether a freshwater mollusk is the intermediate host.^1,2^ This disease is endemic in Brazil, however in the south only northern Paraná is considered an area of endemic transition.^1^ It is suggested that people living in non-endemic areas with little exposure to *S. mansoni* are more susceptible to the development of myelitis from this parasite.^2^ In this context the schistosomal myeloradiculopathy (SMR) is the main ectopic manifestation of this species.^2,3^ The diagnosis of SMR is based on neurological symptoms of spinal cord injury, exams that indicate agent infection, and the exclusion of other causes.^3^

The treatment of SMR can be done with schistosomicides, corticosteroids and/or surgery, however there is no consensus on the effectiveness of one over the other.^3^ Schistosomicides destroy the adult worm and, consequently, interrupt egg production, reducing the inflammatory reaction in the central nervous system (CNS).^4^

This study aims to report a case of schistosomal myeloradiculopathy in a non-endemic area in order to promote early diagnosis and treatment.

## CASE DESCRIPTION

An 11-year-old male patient, weighing 26 kg, previously healthy, was admitted in a pediatric hospital with an acute history of strength loss in the lower limbs one day before admission, with preserved sensitivity. Initially, the patient had a normal cranial computed tomography (CT) scan and cerebrospinal fluid (CSF) analysis. Guillain-Barré syndrome was suspected and immunoglobulin was administered (2g/ kg) for four days, without improvement. Subsequently, a new CSF was collected, which showed a protein concentration of 994 mg/dL, a leukocyte count of 1,845/mm^3^ (49% eosinophils, 89% polymorphonuclear, 11% monocytes) and a glucose concentration of 24 mg/dL. He also had serum eosinophilia (948/μL). Due to the significant increase in serum and CSF eosinophils, the patient received albendazole for five days as an empirical treatment for eosinophilic meningitis. Ceftriaxone and acyclovir were also started empirically. Ten days after the onset of the condition, he lost strength in his left upper limb.

**Figure 1:**
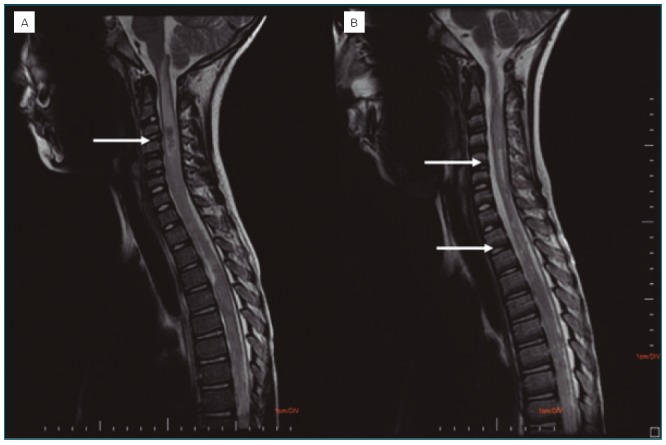
(A) MRI of the cervical spine at the time of diagnosis. The T2-weighted image shows an infiltrative formation with a tumefactive effect. (B) Control cervical spine MRI made after three months shows a reduction in swelling, the appearance of irregularities and tapering areas, and increments of intramedullary cystic degeneration foci.

**Figure 2:**
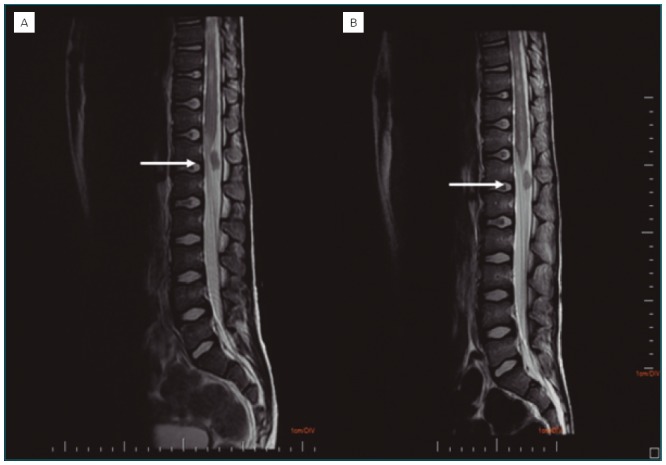
(A) Magnetic resonance imaging of the lumbar spine at the time of diagnosis. The T2-weighted image shows intradural and extramedullary oval formation that promotes displacement of the spinal cord. (B) A control MRI after three months shows no significant changes from the initial exam.

Eleven days after the onset of the symptoms, the patient was transferred to the Pequeno Príncipe Hospital in the city of Curitiba, Paraná, for a neuroaxis nuclear magnetic resonance imaging (MRI). He had a previous history of swimming in a river in the metropolitan region of Curitiba (Colombo). The neuroaxis MRI demonstrated significant medullary canal demyelination, medullary cone enlargement in the thoracolumbar region, in addition to a granulomatous lesion and medullary extrinsic compression in the lower lumbar region (Figures 1 and 2). Serology was then collected for Epstein-Barr virus, cytomegalovirus, human T-cell lymphotropic virus (HTLV), human immunodeficiency virus (HIV) and hepatitis B, in addition to a screening for hypovitaminosis. They were all negative. Two parasitological stool samples were collected, with negative results.

In the first week of hospitalization, the patient developed abnormal lower limb sensitivity and lost strength in his upper limbs. On examination, he had zero grade strength in his lower limbs and four grade strength in his trunk and upper limbs, patellar areflexia, and an abnormal cutaneous sensitivity and proprioception in his lower limbs. In the second week of hospitalization, the patient presented flacid tetraparesis with C6 medullary section level and urinary retention. In this context, correlating the exams from the previous hospital, the fast-evolving clinical situation, the negative serologies and the MRI, an investigation began on the causes of eosinophilic meningitis. Thus, new CSF and serum exams were collected. In these, CSF eosinophils were not found and serum eosinophilia had decreased. However, the patient’s clinical situation remained the same. The new CSF was sent to the *Pontifícia Universidade Católica do Rio Grande do Sul* (PUC-RS) laboratory and the new serum tests were sent to the *Laboratório Central do Estado do Paraná* (LACEN-PR) and to the Fundação Oswaldo Cruz (Fiocruz) in Belo Horizonte, Minas Gerais. The investigation included screening for several agents that are responsible for eosinophilic meningitis, including *Toxocara canis* and *Angyostrongilus*, the latter being considered the main agent of this condition in Brazil. Due to the clinical suspicion of toxocariasis, thiabendazole was prescribed for three days.

Although the patient is not in an endemic region, the clinical features and the MRI findings were compatible with SMR. While waiting for the serological tests, the patient received pulse therapy with methylprednisolone 30 mg/kg for three days, both for the possibility of acute disseminated encephalomyelitis and parasitic infection involving the CNS.

Of the exams collected for eosinophilic meningitis, the only one that was positive was the ELISA method for the detection of IgG antibodies for *Schistosoma mansoni*, a highly sensitive and specific test. Thus, after the result, the patient was treated with praziquantel 60 mg/kg and continued with oral corticosteroid therapy with prednisolone 30 mg/day, according to the Ministry of Health protocol. Furthermore, he received physical therapy for rehabilitation.^3^ By the fourth week, the neurological physical examination showed motor strength and sensitivity corresponding to a C7-C8 medullary section level.

During hospitalization, the patient started with neuropathic pain associated with right-hand paresthesia, which had significant improvement with the association of amitriptyline and gabapentin. About one month after treatment with praziquantel, the dose was repeated because its efficacy is diminished by concomitant corticosteroid therapy.^1^

After two months of the first dose of praziquantel and the start of oral corticotherapy, the patient has grade 4+ strength in the proximal upper limbs, grade 4- strength in the distal upper limbs, grade 1 strength in the lower limbs, and adequate muscular tone. His osteotendinous reflexes are 2+/4+ in his upper limbs and 1+/4 + in his lower limbs, with an absent Achilles reflex. He has vibratory sensitivity preserved up to the proximal region of his right lower limb and up to his left knee. His painful sensory level is at T6. Proprioception is present in his upper limbs and absent in his lower limbs.

At the moment, the patient is taking 30 mg/day of prednisolone and remains hospitalized due to his dependence on a long-term urinary catheter for diuresis and in order to continue rehabilitation with daily physical therapy.

## DISCUSSION

Schistosomiasis is caused by a helmith of the *Schistosoma* family with man being the most important epidemiological host and the freshwater mollusk of the genus *Biomphalaria,* its intermediate host. The south of the country is not an endemic region, except for northern Paraná, which is considered to be an endemic transition area.^1^

*S. mansoni* is the most related to myeloradiculopathy cases, and its natural habitat is in South and Central America.^1,3^ The transmission of the disease depends on the infected host eliminating helminth eggs in its feces. It also depends on intermediate hosts releasing the infected larvae in water collections that do not have adequate basic sanitation. The *S. mansoni* is a tributary venules portal systems guest and can migrate around the body through anastomosis.^1^

Schistosomiasis can be divided into early and late stages.^1^ The initial phase is subdivided into acute asymptomatic and symptomatic forms. The asymptomatic acute form usually occurs in childhood and may go unnoticed or be found in routine exams (eosinophilia and eggs of *S. mansoni* in the stool).^1^ The acute symptomatic form occurs 24 to 72 hours after being infected, with the appearance of erythematous and itchy micropapules, known as cercarian dermatitis.^1^ A differential diagnosis with contact eczema and strobilous prurigo is difficult, when taking into account clinical and epidemiological histories. Still in the acute symptomatic form, symptoms such as lymphadenopathy, malaise, fever, hyporexia, dry cough, sweating, myalgia, abdominal pain, hepatomegaly, splenomegaly, diarrhea, tachycardia, hypotension, headache and malaise may indicate the condition called Takayama fever or a toxemic form. Regarding the symptoms mentioned, their intensity depends on the parasite load and the patient’s sensitivity, and when it worsens, it means that the onset of oviposition (between the fifth and sixth weeks of infection) has begun. The clinical signs and symptoms of the acute symptomatic phase can last 90 days, which make it look like a fever of undetermined origin. The clinical diagnosis is made around 45 days after the infection, through the stool sample or liver biopsy.^1^ Untreated clinical cases usually have spontaneous remission.^1^ At this stage, the disease rarely leads to death, and clinical improvement leads to normalization of body temperature and the disappearance of suggestive symptoms.^1^

In the late phase there are chronic forms, classified according to the most affected organ.^1^ Patients who respond well in the acute phase, meaning fewer inflammatory cells in the granuloma, develop hepatic or hepatointestinal forms. Those who do not respond well, continue having a large granuloma and evolve into the hepatosplenic form of the disease. In the hepatointestinal form, which is frequent in endemic areas, there are no symptoms and the diagnosis becomes accidental with the parasitological stool test. When there are symptoms, they are non-specific and difficult to differentiate from other parasites. On physical examination, there may be pain in abdominal palpation and hepatomegaly without splenomegaly.^1^ A liver biopsy does not usually provide information, but an intestinal biopsy may reveal viable *S. mansoni* eggs.^1^ In the hepatic form, the patient is asymptomatic or has symptoms that are similar to the hepatointestinal form. There are hepatomegaly and liver fibrosis, but no splenomegaly or esophageal varices.^1^ Three main forms can be found in hepatosplenic schistosomiasis: compensated, decompensated and complicated. The compensated form, associated with Symmers fibrosis, has portal hypertension with or without digestive bleeding from the esophageal varices. In children there may be no portal hypertension. In the decompensated form, with a decreased liver function, ascites, jaundice and encephalopathy occur often. Finally, the complicated form is associated with other diseases, such as enterobacterial infections, other liver diseases or other clinical forms of the disease. Ultrasonography improves the diagnostic accuracy of the hepatosplenic form, especially in endemic areas, where individuals may present splenomegaly from other etiologies.^1^

Several other possible clinical forms are possible, such as hypertensive and cyanotic vasculopulmonaries, or pulmonary hypertension from vascular obstruction caused by dead eggs or worms, or by immunocomplex pulmonary vasculitis, all of which are manifested by *cor pulmonale*. Another clinical feature is glomerulopathy, found in 10 to 15% of the cases, and most commonly showed by nephrotic syndrome. Neurological forms from the deposition of the eggs and granulomas in the CNS are also a possibility, with transverse myelitis being the most frequent lesion; the female genitals, testicles, skin, retina, thyroid, and heart are also related but uncommon sites. Pseudoneoplastic lesions, in which tissue reaction suggests bowel tumor formations and lymphoproliferative disease, characterized by non-Hodgkin’s nodular splenic lymphomas and found in 0.9% of 863 splenectomies, are also related as clinical forms.^1^

The central nervous system, especially the spinal cord, is the most related ectopic *S. mansoni* infection.^2^ SMR occurs most in the acute and chronic intestinal forms of the worms, and has a preference for males.^3^ The displacement of eggs and worms to the CNS can be explained by the migration through the Batson epidural venous plexus.^3^ This plexus connects the portal system and the vena cava to the spinal canal veins, reaching the CNS and causing myeloradiculopathy. The disease may initially present a triad of lower back pain, altered lower limb sensitivity, and a urinary disorder, progressing to lower limb weakness and sexual impotence.^3,5^ Clinical manifestations appear in the acute or subacute forms, and it worsens by the second week.^3^

The diagnosis is based on neurological symptoms of spinal cord injury, exams that demonstrate the agent infection, and the exclusion of other causes of myelopathy. The neurological examination shows injury areas of the affected spinal cord. Flaccid paraplegia associated with hyporeflexia, urinary retention and reduced sensitivity demonstrate involvement of the medullary cone and the equine tail. On the other hand, spasticity, altered sensitivity at segmental level and urinary incontinence demonstrate higher spinal cord involvement.^3^ Early recognition is essential in order to avoid irreversible neurological sequelae.

Schistosomiasis diagnostic methods can be differentiated into: Direct diagnostic methods, which detect the parasite or its parts; and indirect diagnostic methods, which identify indirect evidence of the parasite, which depend on biochemical or immunological markers.^6^ The direct diagnostic method recommended by the Ministry of Health is the search for *S. mansoni* eggs in the feces, using Kato-Katz technique which allows for visualization and egg count in the sample, providing a simple and practical quantitative indicator.^1,7^ In the majority of reported SMR cases, *Schistosoma* was found in stool, urine or tissue samples such as the rectal mucosa. ^3^

Among indirect methods, immunological tests are the most used, but have limitations due to cross-reactions with other helminths. In addition, they do not define the intensity of the infection and may remain positive even after it is healed.^3,6,7^ In this context, indirect immunoassays, that is, those that detect host immune response to antigen, are the most widely used, especially indirect hemagglutination, indirect immunofluorescence and the immunoenzyme technique (ELISA).^6^

The indirect hemagglutination reaction is highly sensitive. However, due to cross-reactions with other helminths and inconsistent standardization of the reagents, their reproducibility is impaired.^6,7^ The indirect immunofluorescence reaction is based on the binding of immunoglobulins on parasitic surfaces and fluorescein-labeled human anti-immunoglobulins, which become fluorescent under a microscope.^6^ This method has proven to be practical, inexpensive, and has good sensitivity and specificity detecting IgM antibodies in mild infections.^7^ Among immunological techniques, the ELISA method is very stable and economical, and uses enzyme-linked antigens or antibodies that detect antibodies or antigens.^6^ This technique is the most widely used today, however some authors have observed that the immunofluorescence technique demonstrated higher sensitivity, specificity and predictive value when compared to ELISA.^7^

Ferrari reported two cases in which the CSF analysis of SMR patients revealed nonspecific characteristics: A slight protein increase in 95% of the cases, normal glucose levels and pleocytosis with lymphocyte predominance in 91%. He also found eosinophils in 41% of the cases, an alteration that is compatible with the reported patient. In addition, the identification of anti-*Schistosoma* antibodies using the ELISA, indirect immunofluorescence or hemagglutination techniques had positive schistosomiasis-specific immunological reactions in 85 to 90% of the CSF tested.^3^ Currently, the indirect immunofluorescence reaction is considered to have the best sensitivity and specificity for neuroschistosomiasis.^9^

The MRI showed abnormalities in practically all of the cases of SMR in which it was used, even in cases where myelography or computed myelotomography revealed no abnormality.^5^ MRI has low specificity regarding the differentiation between etiologies: infectious, inflammatory, ischemic, tumoral, swelling or gliosis lesions. On the other hand, it has high sensitivity, which has been strengthening the clinical diagnosis of SMR. ^5^

The treatment of SMR can be performed with schistosomicides, corticosteroids and/or surgery, however there is no study that proves the superiority of one method over another.^3^ In this context, schistosomicides, by destroying the adult worm, interrupt egg production and prevent the inflammatory reaction in the CNS.^4^ The SMR Manual by Brazil’s Ministry of Health recommends praziquantel as the schistosomicidal treatment, at a dose of 50 mg.kg^-1^ in adults and 60 mg.kg^-1^ in children up to 15 years of age, divided into two doses and in association with prolonged oral corticosteroid therapy.^3^ There is no consensus regarding the length of steroid treatment in the literature, however there is evidence that it should be done for more than two months and, if discontinued before six months increases the risk of symptom relapse.^3,10^ Badr, in a report of 17 patients with SMR in Egypt, reported that among patients who discontinued the corticosteroids early on (less than 60 days of use), 75% had recurrent symptoms of myelopathy.^10^ Andrade, analyzing 16 SMR patients treated with praziquantel 60 mg/kg/day for three days in combination with prednisone 100 mg/day, showed that improvement in the symptoms was observed starting from the first two weeks of treatment. The regression speed varied according to each patient.^11^

Surgery should be reserved for patients with acute paraplegia and blocked CSF, and for those whose clinical condition worsens despite conservative treatment. It should be used less frequently for diagnostic purposes.^3^

SMR is a serious condition that has a risk of major neurological sequelae. However, it is underreported due to the difficulty of clinical recognition and the limitations of diagnostic tests.^3^ In our country, underreporting is even worse, because our state is not considered to be an endemic region. The clinical features associated with the laboratory tests that suggest the presence of the causative agent, and an MRI with suggestive images, help corroborate the diagnostic hypothesis of SMR. Early treatment of parasitic infections involving the CNS improves the prognosis and decreases morbidity and mortality.^10^

Importantly, the occurrence of SMR, unlike other severe forms of schistosomiasis, does not depend on high parasitic loads. Generally, patients with SMR have few eggs per gram of feces and often come from low prevalence areas. These findings justify the implementation of epidemiological surveillance of SMR throughout the country, even in non-endemic states, since people can migrate freely throughout the national territory, including those who carry *S. mansoni*. ^3^

This case study was made to enlighten health professionals about this condition and advise them on the importance of considering a different diagnosis after empiric treatment fails, even if the diagnosis is outside the norm.
